# Acupuncture and its role in the treatment of ischemic stroke: A review

**DOI:** 10.1097/MD.0000000000039820

**Published:** 2024-10-04

**Authors:** Zuoshan Wang, Manya Wang, Haishen Zhao

**Affiliations:** a Helen Hospital of Traditional Chinese Medicine, Suihua City, Heilongjiang Province, China; b Shanghai Pudong New Area Nanhui Xincheng Community Health Service Center, Pudong New Area, Shanghai Province, China.

**Keywords:** acupuncture, ischemic stroke, meta-analysis, randomized controlled trials, research progress

## Abstract

Acupuncture is a traditional Chinese medicine therapy that is treatment by placing a needle or pressure in a specific position on the patient’s skin. Although used in the treatment of various diseases, acupuncture is effective in the treatment of ischemic stroke (IS), and has made some progress in the mechanism of action of the treatment of this disease. IS is difficult to treat, and there is a high rate of disability. Drug therapy is usually the first line of treatment, but adjuvant therapy has outstanding efficacy in promoting the rehabilitation of the disease and preventing sequelae. Among them, acupuncture is getting more and more attention as a more popular treatment method. Therefore, this study excavates the high-quality randomized controlled trials and meta-analysis of acupuncture for IS in recent years to further summarize the efficacy of acupuncture for IS. In this review, we provide an overview of the current understanding of acupuncture and IS, and the current studies investigating the effectiveness of acupuncture in the treatment of IS.

## 1. Introduction

Ischemic stroke (IS) is a kind of cerebrovascular disease characterized by ischemic necrosis or encephalomalacia of localized brain tissue, which is caused by the stenosis or occlusion of cerebral blood supply arteries. It is the second cause of death in the world. IS has the characteristics of high morbidity, high disability rate, high mortality rate, high recurrence rate, and high economic burden.^[1]^ The Chinese population has a high incidence of stroke, about 80% of which is IS. The one-year mortality rate of IS patients treated in Chinese hospitals is 14.4% to 15.4%, and the mortality/disability rate is about 33.4% to 33.8%.^[2]^ At the same time, according to the American Heart Association’s Heart Disease and Stroke Statistics Report in 2017, data from a national cohort study of 18,462 participants showed that the prevalence of stroke-related symptoms was found to be at least 1 stroke-related symptom in 17.8% of the population aged >45 years, and the prediction showed that the incidence of stroke-related symptoms was rising. About 87% of stroke patients in the United States have IS.^[3]^ At present, the treatment of IS is mainly through drug thrombolysis or mechanical thrombectomy to improve cerebral blood circulation, restore the blood flow state of blocked blood vessels as soon as possible, ensure the stability of cerebral blood flow, and reduce nervous system injury. At the same time, reducing the incidence of hypertension, hyperlipidemia, diabetes, and other high-risk factors in the population can also indirectly reduce the incidence or recurrence rate of IS.

Acupuncture has a prominent effect on the treatment of IS, because acupuncture may effectively improve the injury after stroke, in which acupuncture plays an important role in improving the perfusion around the infarction, increasing the perfusion of the low perfusion area of the affected lobe, and promoting neuronal reorganization, as well as rehabilitation treatment in the middle and late stages of stroke.^[4]^ This article will discuss and analyze the acupuncture treatment of IS, and review its mechanism of action.

## 2. Acupuncture

In traditional Chinese medicine (TCM) theory, acupuncture is an operation in which instruments (metal needles) are inserted into specific locations (acupoints) of the human body. It is an ancient form of healing with a long history of prosperity. Acupuncture and moxibustion, first recorded in the Yellow Emperor’s Canon of Internal Medicine, is an important part of TCM. It has the characteristics of wide application, obvious curative effect, convenience and economy, and has been used for thousands of years. Acupuncture is the treatment of disease by activating specific areas on the patient’s body called acupuncture points. When these points are fully activated, the patient or acupuncturist will experience a feeling of pain, numbness, fullness, or heaviness called Deqi or Taqi.^[5]^ According to the theory of TCM, Qi is the most important internal energy of the human body, and the balance of Qi is essential to the health of the body. If Qi is out of balance, diseases will occur. The main mechanism of acupuncture and moxibustion is to give special stimulation to acupoints to restore the balance of Qi.^[6]^ The main guiding theory of acupuncture and moxibustion is the meridian theory, which studies the physiological functions and pathological changes of the human body and their relationship with the viscera. The meridians and collaterals belong to the zang-fu organs and reach the joints of the limbs. They integrate the zang-fu organs, tissues, and other organs into an organic whole, transport Qi and blood through the meridians and collaterals, regulate yin and Yang, and coordinate the functions and activities of all parts of the body.^[7]^ Qi of the body flows through these meridians and participates in the homeostatic regulation of various functions of the body to maintain the health of the body.^[8]^

Acupuncture is widely accepted and applied in the world because of its safety, effectiveness and convenience. Clinical research provides a scientific basis for clinical decision-making. At the same time, basic research has also explained its internal mechanism. With current technology, acupuncture can be studied with functional magnetic resonance imaging (fMRI), which also provides support for a direct link between acupuncture points and specific brain activities. Numerous studies have analyzed the significant advantages of acupuncture for stroke and post-stroke spastic hemiplegia, migraine, musculoskeletal pain, and peripheral neuropathy.^[9]^ The World Health Organization recommends acupuncture as an alternative and complementary strategy for the treatment of stroke, and relevant clinical trials and meta-analyses have proved that acupuncture has a good effect on improving the balance function of stroke patients, reducing muscle spasm and increasing muscle strength after stroke.^[10]^ There are 2 main forms of acupuncture, electroacupuncture and manual acupuncture, which are widely used in the clinical treatment of a variety of diseases.

## 3. Ischemic stroke

### 3.1. Epidemiology

Stroke is the second leading cause of disability and death globally and one of the highest financial burdens in low- and middle-income countries. From 1990 to 2019, the total number of strokes increased by 70.0%, the prevalence increased by 85.0%, the mortality rate increased by 43.0%, and the disability rate increased by 32.0% due to stroke.^[11]^ At the same time, a global statistics conducted in 2016 found that there were 13.7 million new stroke events, about 87% of which were IS, while only about 5% of IS patients in the world could be treated within the qualified treatment time window.^[12]^ According to China Stroke Report 2020, the annual incidence of stroke in China is 276.7/100,000, and the prevalence rate is 2022.0/100,000, which has become the leading cause of death and disability among Chinese residents.^[13]^ Acute onset and rapid progress of stroke can lead to limb paralysis, language disorders, dysphagia, cognitive impairment, depression, and so on, which seriously affect the quality of life of patients and bring huge economic burden to patients, families, and society. It is estimated that the cost of stroke has exceeded 721 billion US dollars, accounting for 0.66% of global GDP.^[14]^ In 2019, 1853 tertiary hospitals in China received about 3 million stroke patients in a year, and the average medical cost of each stroke patient was about 20,000 yuan.^[15]^ However, IS is largely preventable. Modern findings suggest that the risk of 90% of patients with IS is associated with the following 10 risk factors (history of hypertension, smoking, waist-to-hip ratio, dietary risk score, regular physical activity, diabetes, alcohol intake, psychosocial stress, and depression, cardiac causes, and ratio of apolipoprotein B to A1). Among them, hypertension, smoking, waist-to-hip ratio, diet, and alcohol consumption are important risk factors for IS. Therefore, controlling blood pressure and reducing smoking, promoting physical activity and healthy diet are targeted interventions that can greatly reduce the risk of IS.^[16]^

### 3.2. Pathophysiology

IS is a kind of cerebrovascular disease caused by various reasons, which leads to focal neurological deficit or central nervous system injury. The injury outcome of IS depends largely on the amount of ischemia-related neuronal death in the affected brain region. Ischemia/reperfusion (I/R) of brain tissue can lead to biological energy failure, loss of cellular ion homeostasis, excitotoxicity, mitochondrial dysfunction, reactive oxygen species (ROS) generation, and other pathogenic pathways, which are considered to be the core mechanism of neuronal death in IS. At present, there are many theories about the pathogenesis of IS, including excitatory amino acid toxicity theory, inflammatory response theory, oxygen free radical injury theory, nerve cell apoptosis theory, and hemodynamic changes theory.

Excitatory amino acid (EAA) toxicity is now considered to be the main cause of secondary injury after cerebral ischemia. Experimental studies have shown that EAA is involved in the pathogenesis of ischemic neuropathy. At the same time, ischemia and hypoxia of brain tissue will lead to the failure of energy metabolism in normal brain tissue, and then the release of EAA and excitatory amino acids will activate N-methyl-D-aspartate receptors, which will lead to the entry of calcium and the activation of downstream cell death signals, thus leading to the abnormal activation of many calcium-dependent enzymes in the brain. Initiates necrosis, apoptosis, and autophagy, thereby causing vasoconstriction and impairing the blood–brain barrier (BBB), and one of the hallmark pathophysiological features of IS is BBB disruption.^[17–19]^

Ischemia induces neuroinflammation by promoting the production of proinflammatory mediators that induce cell death and cell dysfunction, resulting in activation of glial cells and infiltration of peripheral leukocytes. Neuroinflammation can reduce tissue damage by removing dead cells and debris; however, excessive inflammation is harmful due to the production of neurotoxins and brain edema.^[20]^ Studies have shown that glial cells can regulate neuroinflammation after stroke. Glial cells can integrate the signals of neuronal damage, release cytokines, attract immune cells to the stroke site, and interact with other immune cells and affect their condition.^[21]^ Immediately after IS, neurons and glial cells at the site of injury activate astrocytes by releasing products such as damage-associated molecular pattern molecules. Reactive astrocytes secrete proinflammatory cytokines, chemokines, and matrix metalloproteinases, which subsequently disrupt the BBB and recruit leukocytes from the peripheral blood, resulting in secondary brain tissue injury.^[22]^ After this activation process, microglia release a large amount of proinflammatory cytokines (TNF-α, IL-6, and IL-1β), which are considered to be the main factors involved in the acute inflammation of IS.^[23,24]^

Oxidative stress (OS) is an important pathophysiological response to ischemic cerebrovascular disease, which runs through all stages of cerebral ischemia and is one of the main mechanisms of acute cerebral ischemic injury. When cerebral ischemia occurs, the blood circulation in the brain is impaired, and a large amount of ROS is produced in the brain. ROS is related to brain edema, calcium overload, mitochondrial dysfunction, excitotoxicity, iron release, and inflammation. Moreover, the increased ROS can activate matrix metalloproteinases, thereby increasing the permeability of BBB, allowing harmful substances in the body to enter the brain and aggravating the damage to brain tissue.^[25]^ Meanwhile, the reperfusion treatment of IS can also promote the production of ROS, and OS is one of the main reasons for most of the ischemia–reperfusion injury of brain tissue.^[26]^ At the same time, ROS produced after cerebral ischemia can activate a series of inflammatory factors to induce inflammatory response. Moreover, microglia, astrocytes, and related immune responses in inflammatory response can also promote the production of ROS and aggravate OS, thus aggravating local tissue damage.^[27]^ However, further studies have shown that ROS produced by ischemia can activate hypoxia-inducible factors and downstream pathways, while hypoxia-inducible factors and downstream pathways in the late stage of ischemia can promote the differentiation of neural stem cells, neurogenesis, nerve migration and other functions, and ROS are involved in tissue repair in the late stage of ischemia.^[28]^

Neuronal apoptosis is the mechanism of nervous system damage in I/R injury. In I/R injury, it exists both in the initial hypoxia stage and in the reperfusion state. In the hypoxia stage, the intrinsic pathway plays a more important role. Hypoxia causes mitochondrial damage, leading to the formation of apoptotic bodies and the activation of caspase 9, which in turn leads to the activation and execution of caspase 3 pathway. In the reperfusion state, abundant inflammatory mediators are responsible for the activation of extrinsic pathways, in which caspase 8 activation leads to caspase 3 activation as well as the execution of pathways, the formation of apoptotic bodies and cell death.^[29,30]^

At present, many scholars believe that the pathogenesis of IS is related to vascular obstruction caused by changes in cerebral hemodynamics, hemorheology, and cerebral vascular structure or morphology. Generally speaking, the cerebral blood circulation of normal people is maintained in a relatively balanced and stable state, but when the internal carotid artery and vertebral artery are narrowed or occluded, or the cerebral blood supply is insufficient, it will lead to a decrease in cerebral blood perfusion. When the cerebral perfusion is insufficient to maintain normal brain function, it will lead to ischemia and hypoxia of brain tissue, leading to the occurrence of IS.^[31]^ When the blood supply vessels of the brain are narrowed or occluded, in order to maintain the blood supply of the brain, it will inevitably affect the hemodynamic parameters such as blood flow velocity, perfusion pressure, vascular resistance, and so on. The maintenance of cerebral perfusion in the early stage of stenosis or occlusion depends on the establishment of collateral circulation and the expansion of small resistance vessels. However, when the supply of the 2 cannot meet the needs of cerebral metabolism, the resistance of small vessels reaches extreme expansion, and cerebral ischemia is further aggravated, which can lead to the occurrence or progression of IS.^[32]^

### 3.3. Diagnosis

Patients with an acute episode of focal neurologic deficit should receive a quick, focused history and examination. The medical history should include the date of the patient’s last visit, details of the onset, risk factors, medications, and clues to possible related conditions. Non-contrast head computed tomography (CT) is the preferred study. Head CT may be normal early in the onset of acute ischemic stroke (AIS), and many centers often add angiography and CT angiography to the initial CT study in order to plan for possible emergency thrombectomy. CT angiography from the aortic arch to the corona allows the identification of large vessel occlusions that may be suitable for endovascular treatment. Magnetic resonance imaging (MRI) is more sensitive for early identification of AIS, however, it is not often used as an initial study because it is less extensive and takes longer to complete. The sensitivity of diffusion-weighted imaging and apparent diffusion coefficient (ADC) for acute cerebral infarction was close to 100%. MRI lesions bright on diffusion-weighted imaging, dark on ADC, no early changes magnetic resonance angiography can demonstrate blood flow or stenosis or occlusion in the thoracic, cervical, and cephalic arteries. Perfusion imaging to identify the core and penumbra can be performed using CT or MRI in many cases where late presentation and endovascular thrombectomy are considered. Diagnostic digital subtraction angiography remains the gold standard for angiography and should be performed urgently before endovascular thrombectomy.^[33]^

### 3.4. Traditional treatment of IS

The primary therapeutic goals of IS are revascularization and limitation of secondary neuronal damage. Therefore, the treatment of patients with AIS at home and abroad is mainly through timely reperfusion to restore cerebral blood flow, save ischemic tissues, and reduce disability rate. The efficacy and safety of intravenous thrombolysis in patients with AIS within 4.5 hours of onset have been fully confirmed. In patients with acute intracranial arterial occlusion, endovascular therapy (EVT) plus intravenous thrombolysis can significantly reduce the disability rate, but in clinical practice, about 25% of patients arrive at the emergency room within 4.5 hours after onset. Only 3% to 9% of AIS patients receive intravenous recombinant tissue plasminogen activator, and most AIS patients often miss the opportunity to receive recombinant tissue plasminogen activator intravenous thrombolytic therapy.^[34–36]^ patients with AIS of proximal large vessel occlusion who have been evaluated by rigorous imaging for more than 6 hours can be treated by EVT. Because of EVT, the time window of AIS treatment has been expanded, and it provides a more powerful treatment for doctors.^[37]^ With the implementation of effective reperfusion strategies, intravenous thrombolysis and EVT have become the most common treatments for patients with IS. However, these therapeutic strategies have a relatively narrow thrombolytic time window (≤4.5 H), low thrombus targeting ability, and the risk of hemorrhagic transformation. In addition, the restoration of blood flow to the ischemic brain leads to secondary reperfusion injury, and reperfusion intensifies the production of ROS and the amplification of inflammatory and immune responses, leading to excessive neurologic death. It impairs the integrity of the BBB and ultimately leads to brain edema, which is also the limiting point of the above therapy.^[38]^ Neuroprotective agents can act on different pathophysiological pathways of IS, thus exerting neuroprotective effects at different stages of stroke. One of the main therapeutic goals is to prevent glutamate-mediated excitotoxicity. Secondly, eliminating OS is also a potential therapeutic strategy to rescue ischemic neurons. In addition, current studies have focused on attenuating the immune-mediated inflammatory response in the acute phase of IS. Neutrophils play an important role in the occurrence and development of inflammation. E-selectin antibody therapy can reduce the infiltration of neutrophils into the ischemic brain and reduce the infarct volume.^[39–41]^ Current emerging stem cell therapies have emerged as attractive candidates for cell therapy in the treatment of stroke. Stem cells have multiple beneficial effects, including neuroprotection, reduced inflammation and immune response, and increased angiogenesis and neurogenesis, but the primary mechanisms by which each cell type is able to mitigate the damage caused by stroke have not been elucidated. Therefore, before cell therapy becomes a standard stroke treatment, it is necessary to coordinate basic science and clinical cooperation to further clarify its mechanism of action.^[42]^

## 4. Understanding of IS in TCM

According to TCM theory, IS belongs to the category of stroke. The main pathogenesis of apoplexy is the imbalance of heart, liver, and kidney, the imbalance of yin and Yang of the body, and the disorder of Qi and blood, which leads to a common and frequently-occurring disease of cerebral stasis. Clinically, the main symptoms are sudden fainting, limb discomfort, mouth and tongue deviation, speech discomfort, and limb numbness. In the ancient Chinese book Huangdi Neijing Suwen, it is recorded that “the Yu of the 5 zang-organs and 6 fu-organs in the wind is also the wind of the zang-organs, and when each enters its portal, it is the partial wind..” If the wind follows the wind, it is the brain wind. And “When blood and Qi go together, it is a great syncope, and a syncope leads to sudden death; when Qi returns, it leads to life; when it does not return, it leads to death.” It profoundly describes the etiology and pathogenesis of IS. Moreover, Zhang Xichun, an expert in TCM in the Qing Dynasty, wrote in his book Yi Xue Zhong Zhong Shen Xi Lu: “Dongyuan said that if a person’s vitality is insufficient, the evil will gather together and make him fall down and stiff, like the wind..” The blood of the wife’s body is originally prevalent with Qi, and the rise of Qi is too much, which can cause congestion in the brain and squeeze out the nerves of the brain. The pathological mechanism described is highly consistent with the understanding of the pathological mechanism of “IS” in modern medicine.

With the further study of molecular biology in modern medicine, it has been gradually recognized that TCM has the functions of anti-inflammatory, anti-OS, inhibiting apoptosis, repairing damaged blood-brain barrier, promoting neuronal regeneration and angiogenesis, and plays a key role in the occurrence and development of stroke. Many active ingredients of TCM have shown significant therapeutic effects in pharmacological experiments and clinical applications. The pathological environment after cerebral ischemic injury is not conducive to endogenous nerve repair. Therefore, TCM can improve the microenvironment, inhibit apoptosis and gliosis, improve the pathological damage of neurons and promote the recovery of neurovascular units by promoting the release of nutritional factors and inhibiting inflammation.^[43]^ At the same time, studies have found that TCM can effectively inhibit apoptosis, which is of great significance for alleviating I/R injury.^[44]^ In addition, acupuncture also plays an important role in the treatment of IS. Acupuncture can improve the neurological deficits caused by IS, especially dyskinesia, spasm, cognitive impairment and dysphagia. Studies have found that acupuncture plays a key role in regulating neural plasticity, and its mechanism of action is to stimulate the regeneration of nerve cells, promote the replacement of functional cells that die after stroke, and lay a structural foundation for the establishment of contacts and synapses between cells by promoting the growth of new nerve processes or the regeneration of damaged axons. Thereby ultimately facilitating information transfer by enhancing the structural connectivity and transmission of individual synapses, neural networks, and brain regions, leading to neuroplasticity-mediated structural reconstruction and functional recovery.^[45]^ At present, although the mechanism of action of some TCM in the prevention and treatment of IS has been confirmed, it still needs further systematic research and improvement. TCM pays attention to the overall regulation, syndrome differentiation and treatment of patients with IS, and uses acupuncture and moxibustion to cooperate with treatment, which has achieved remarkable results.

## 5. Evidence of acupuncture and moxibustion for IS

Acupuncture is currently recommended by the World Health Organization (WHO) as an alternative and complementary strategy for the treatment and improvement of the clinical symptoms of IS. Clinical studies have found that acupuncture and moxibustion have good advantages in improving brain tissue injury after IS, especially in analgesia, limb rehabilitation, improving blood flow around infarction, and stimulating brain lobes and neuronal reorganization in low blood flow perfusion areas.^[4]^ Wang W et al^[46]^ conducted an animal experimental retrospective study on acupuncture intervention in IS model, and the results showed that acupuncture had significant advantages in reducing infarct volume and improving nerve injury in IS rat model, and could play a neuroprotective role in acute ischemia animal model. At the same time, a series of clinical studies and laboratory evidence show that the mechanism of acupuncture on cerebral ischemia is complex and multi-level, including: nerve protection, collateral circulation reconstruction, nerve regeneration, and so on. The neuroprotective effect of acupuncture is mainly through regulating inflammatory factors in brain tissue or peripheral blood, indirectly inhibiting the activation of nuclear factor-κB, and reducing the synthesis and secretion of proinflammatory cytokines. Acupuncture can reduce the accumulation of excitatory amino acids in local infarcted area, thus reducing the toxic effect of glutamate on neurons. Acupuncture and moxibustion can reduce the OS caused by cerebral ischemia. Acupuncture and moxibustion can inhibit apoptosis and improve nerve function injury through PI3K/Akt and extracellular signal-regulated kinase or terminal kinase mediated mechanism.^[47–50]^ Acupuncture plays an important role in the treatment of ischisis, and it has a good effect in all stages of ischisis, and it is also recommended as an alternative and adjuvant therapy in the world.

In this section, we present and discuss the various studies conducted by acupuncture. Table 1 summarizes these studies. This review includes several recent randomized controlled trials (RCTs) and meta-analyses to help formulate conclusions on the safety and efficacy of the use of acupuncture for the treatment of IS.

**Table 1:** Evidence of acupuncture and moxibustion for ischemic stroke.

First author of the study (year)	Study groups and interventions	Results and findings	Conclusion
Chen Wen et al (2006)^[51]^	Participants (n = 160) were equally divided into 2 groups: (1) acupuncture group (2) control group Patients in the acupuncture group received basic treatment plus acupuncture treatment, while patients in the control group received basic treatment.	The ability of cerebral vasomotor reaction, the function of cerebral blood flow autoregulation and the compensatory function of cerebral hemisphere collateral circulation in the acupuncture group were significantly enhanced compared with those before treatment (*P* < .05, *P* < .01), and were superior to those in the control group (*P* < .05).	Acupuncture at Zusanli (ST 36) and Xuanzhong (GB 39) can significantly improve the cerebral vasomotor reaction, cerebral blood flow autoregulation and cerebral collateral circulation compensation in patients with ischemic stroke.
Fanrong Liang et al (2006) [52]	Patients diagnosed with ischemic stroke (n = 290) were randomly divided into 2 groups, one group received Xingnao Kaiqiao acupuncture, and the other group received sham-acupoints acupuncture.	Compared with the sham-acupoints acupuncture treatment, the recurrence rate of the Xingnao Kaiqiao acupuncture group was significantly reduced (*P* < .001); there was a significant difference in NIHSS (4.15 ± 2.032 vs 6.35 ± 3.131, *P* < .01). Six months after treatment, the stroke-specific quality of life scale (166.63 ± 45.70) was higher in the Xingnao Kaiqiao acupuncture group than in the sham-acupoints acupuncture group (143.60 ± 50.24), and the difference was statistically significant (*P* < .01).	The results of this trial showed that the relapse rate was clinically reduced in patients treated with Xingnao Kaiqiao acupuncture at the end of 6 months compared with sham-acupoints acupuncture; Improve self-care ability and quality of life.
Yong Huang et al (2012) [53]	Patients diagnosed with ischemic stroke (n = 43) They were randomly divided into 5 groups: (1) TE5 acupuncture group (2) TE5 sham acupuncture group (3) sham acupoint acupuncture group, (4) sham acupoint acupuncture group, and (5) non-acupuncture group.	Compared with the non-acupuncture group, neither TE5 sham acupuncture nor sham acupuncture/sham acupuncture activated brain regions. However, needling at TE5 resulted in activation of the Brodmann Area; Needling at TE5 activated Brodmann areas 13, 19, and 47 and did not inactivate any area compared to sham needling at TE5; Compared with sham acupuncture, TE5 acupuncture did not activate Brodmann Area 9, but inactivated it.	PET-CT showed that TE5 acupuncture had a modulatory effect on the functional areas of the brain, which may be related to its impact on the rehabilitation of patients after stroke.
Chun-Chuan Shih et al (2015) [54]	Retrospective cohort study of patients with ischemic stroke (n = 30,058), who were equally divided into 2 groups and received no acupuncture or acupuncture after stroke. A propensity score was applied to match age, gender, and key variables to the acupuncture group.	Patients with ischemic stroke had a reduced risk of recurrence in the acupuncture-treated cohort compared with the non-acupuncture-user cohort. The risk of recurrent ischemic stroke was the lowest after drug and acupuncture treatment.	These data provide support for the use of acupuncture in patients with ischemic stroke.
Wu Tu Hsing et al (2012) [55]	Patients diagnosed with ischemic stroke (n = 62) Patients were assigned to either a true scalp electroacupuncture treatment group or a placebo electrostimulation group.	Compared with placebo electrical stimulation, the true scalp EA treatment group had greater functional improvement after 10 scalp EA sessions as measured by NIHSS (*P* = .0023).	These results support further testing of scalp EA for ischemic stroke and further mechanistic studies to understand the mechanisms associated with the observed improvements.
Zhan Daowei et al (2023) [56]	Patients diagnosed with acute ischemic stroke (n = 102) The patients were assigned to the control group (thrombolysis + routine medical treatment) or the observation group (control group + acupuncture treatment on the basis of treatment).	NIHSS, mRS scores and serum Hcy, hs-CRP levels in the observation group were lower than those in the control group (*P* < .05, *P* < .01), and MBI scores were higher than those in the control group (*P* < .01). The total effective rate of the observation group was 88.2% (45/51), which was higher than that of the control group 70.6% (36/51, *P* < .05).	Acupuncture can promote the recovery of neurological function and improve the ability of daily living in patients with AIS thrombolysis, which may be related to reducing the level of inflammatory factors to inhibit inflammatory reaction and improve the reperfusion injury after cerebral ischemia.
Lifang Chen et al (2016) [57]	Patients diagnosed with ischemic stroke (n = 250) The patients were randomly divided into 2 groups: (1) acupuncture group (2) non-acupuncture group.	Compared with the non-acupuncture group, the acupuncture group had a significant advantage in the improvement of the scores of NIHSS (*P* < .001), VFSS (*P* < .001), MMSE (*P* < 0. 001) and MoCA (*P* = 0. 001), and had a lower incidence of mild acupuncture reactions and a better safety.	The trial showed that acupuncture was safe and had additional multiple efficacy in improving neurologic deficits, swallowing disorders, cognitive impairment, and lower extremity function.
Dong Hua, et al (2019) [59]	Patients diagnosed with headache in the recovery stage of ischemic stroke (n = 97) The patients were randomly divided into 2 groups: (1) acupuncture group (n = 57) and (2) western medicine group (n = 40).	Compared with the western medicine group, the changes of VAS score, headache score, plasma pain-related factor content and plasma endogenous opioid peptide content in the acupuncture group were greater than those in the western medicine group (*P* < .05). The total effective rate was 84.2% (48/57) in the acupuncture group and 62.5% (25/40) in the western medicine group (*P* < .01). The recurrence rate in the acupuncture group was lower than that in the western medicine group (*P* < .01).	Acupuncture is more effective than flunarizine hydrochloride in the treatment of headache in the recovery stage of ischemic stroke, and its mechanism may be related to increasing the secretion of EP and reducing the expression of pain-related factors.
LI Wenqian, et al (2022) [63]	Patients diagnosed with acute ischemic stroke (n = 60) The patients were randomly divided into 2 groups: (1) electroacupuncture combined with conventional western medicine therapy (2) simple conventional western medicine therapy.	Compared with the conventional western medicine therapy, the NIHSS score of the patients treated with electroacupuncture combined with conventional western medicine therapy decreased significantly (*P* < .05), and the MBI score increased significantly (*P* < .05). The level of IL-17 was significantly decreased and the level of IL-10 was significantly increased in patients treated with electroacupuncture combined with conventional western medicine (*P* < .05).	Electroacupuncture combined with conventional western medicine can improve the neurological function and activities of daily living in patients with AIS.
YU Nan-nan et al (2021) [64]	Patients diagnosed with ischemic or hemorrhagic stroke (n = 360) Patients were treated with bloodletting at the 12 well points of the hand (n = 180) or standard medical therapy (n = 180).	Compared with the standard drug treatment group, the patients in the blood-letting therapy group had a significantly higher rate of consciousness improvement after 80 minutes of treatment (*P* < .05). In a separate analysis of the subgroup with moderate impairment of consciousness, blood-letting therapy at the 12 well points of the hand was beneficial for patients with ischemic stroke and significantly improved the eye and language responses to GCS scores (*P* < .05).	Blood-letting therapy of hand 12 well points is safe, which can improve the level of consciousness of patients with ischemic stroke, and is a safe emergency intervention measure for acute stroke.
Li Li et al (2014) [65]	Twenty-five RCTs involving 2224 participants diagnosed with ischemic stroke. Participants were treated with acupuncture, sham acupuncture, or medication	The acupuncture treatment group was superior to the control group, with significant difference in clinical efficacy [OR = 4.04, 95% CI (2.93, 5.57), *P* < .001], and Fugl-Meyer assessment [MD = 11.22, 95% CI (7.62, 14.82), *P* < .001]. Barthel index score [MD = 12.84, 95% CI (9.85, 15.82), *P* < .001], and neurological deficit score [MD = −2.71, 95% CI (−3.84, −1.94), *P* < .001].	While most of the results in his analysis supported acupuncture, the study concluded that because the quality of the evidence supporting the findings was low, further research was necessary to verify the findings. The authors do, however, conclude that acupuncture is superior to non-acupuncture or drug therapy in the treatment of ischemic stroke.
Ai-Ju Liu et al (2015) [66]	Eighteen RCTs involving 1411 participants were included. The patients were treated with either fake acupuncture, medication or real electroacupuncture.	The results of meta-analysis showed that the electroacupuncture group was better than the conventional western medicine group in improving Barthel index (*P* < .01), Fugl-Meyer score (*P* < .01), NIHSS score (*P* < 0. 01), and revised Scandinavian Stroke Scale (*P* < 0. 01).	While these findings suggest evidence for EA in acute ischemic stroke, further large-scale RCTs are needed to validate these findings.
Huacong Liu et al (2021) [67]	Thirty patients with dominant MCA AIS were recruited for resting-state functional magnetic resonance imaging (Rs-fMRI) scan They were randomly divided into 2 groups: (1) control group (2) scalp acupuncture group.	Compared with the control group, the scores of NIHSS in the scalp acupuncture group were significantly improved (*P* < .05). VMHC, ALFF and ReHo increased after treatment in the scalp acupuncture group. VMHC increased in the bilateral BA6 and BA8 regions. ALFF of left BA39 and its adjacent superior and middle temporal gyri increased. The ReHo values of left middle temporal gyrus (including BA21) to BA37, left BA40, angular gyrus to BA7 were increased.	The findings suggest that scalp acupuncture treatment can enhance regional brain functional activity and functional connectivity between hemispheres related to sensory integration, language processing, and motor coordination in patients with MCA AIS.

ALFF = amplitude of low-frequency fluctuation, CI = confidence interval, GCS = Glasgow Coma Scale, RCT = randomized clinical trial, ReHo = regional homogeneity, VMHC = voxel-mirrored homotopic connectivity.

The first was a multicenter, single-blind, randomized controlled study RCT in 2006 that used transcranial Doppler ultrasound to investigate the effects of acupuncture on cerebrovascular function in patients with IS. Hemiplegia, hemianopia, sensory disturbance, aphasia, and other clinical symptoms are caused by cerebral blood supply disorders, ischemia and hypoxia, which lead to necrosis and softening of brain tissue. In this study, the authors found that the ability of cerebral vasomotor response, the function of cerebral blood flow autoregulation and the compensatory function of collateral circulation in the cerebral hemisphere in the acupuncture group were significantly enhanced compared with those before treatment (*P* < .05, *P* < .01), and were better than those in the control group (*P* < .05). The results of this study reflect the effects of acupuncture on cerebrovascular function, which may play a role in reducing clinical symptoms in patients with IS.^[51]^

The second was a 2012 RCT that showed the benefit of acupuncture on IS in an intervention group of 145 patients treated with Xingnao Kaiqiao acupuncture and a control group of 145 patients treated with sham-acupoints acupuncture. The clinical trial showed that the number of recurrent cases in the Xingnao Kaiqiao acupuncture group was lower than that in the sham-acupoints acupuncture group (*P* < .001). There was a significant difference in the National Institutes of Health Stroke Scale (NIHSS) between the 2 groups at 4 weeks of treatment (*P* < .01). After 6 months of treatment, the stroke-specific quality of life scale (166.63 ± 45.70) was higher in the Xingnao Kaiqiao acupuncture group than in the sham-acupoints acupuncture group (143.60 ± 50.24; *P* < .01). Treatment results showed a clinically reduced relapse rate in patients with IS treated with Xingnao Kaiqiao acupuncture at the end of 6 months compared with sham acupuncture.^[52]^

The third was a retrospective study of 30,058 patients newly diagnosed with IS between 2000 and 2004, reported in 2015. The authors of this study reported that the acupuncture cohort (n = 15029 patients), after matching demographic variables with a 1:1 propensity score) had a reduced risk of recurrent stroke (HR = 0.88; 95% confidence interval [CI] = 0.84–0.91) in the therapeutic intervention acupuncture treatment compared with the non-acupuncture cohort (n = 15029 patients). The effect of acupuncture was significant in patients who received or did not receive stroke prevention drugs, but its effect decreased with the age of stroke patients. The authors conclude that acupuncture may be effective in reducing the recurrence rate of IS.^[53]^

A 2012 study in which acupuncture modulates glucose metabolism in functional areas of the brain in patients with IS used PET-CT to investigate functional area responses to acupuncture treatment. In this study, the authors found that neither the sham acupuncture at Waiguan (TE5) nor the sham acupuncture/sham acupuncture activated brain regions compared with the non-acupuncture group. However, needling at TE5 resulted in activation of the Brodmann Area (BA) brain region. In contrast to sham needling at TE5, needling at TE5 activated BA13, 19, and 47, which are located in the right hemisphere and are involved in cortical association, visual processing, and motor execution, respectively, and did not inactivate any region. Compared with sham acupuncture, TE5 acupuncture had no effect on BA9 activation, but had effect on BA9 inactivation. BA9 was located in both hemispheres and was involved in the execution of management. The findings of this study reflect the modulatory effect of TE5 acupuncture on functional areas of the brain.^[54]^

A RCTs in 2012 compared real and sham acupuncture to assess the efficacy of scalp subcutaneous electrical stimulation for spontaneous functional recovery in patients with IS. The authors reported greater functional improvement on the National Institutes of Health Stroke Scale UNK5NIHSSUNK6 (mean ± SD: 3.75 ± 2.30 vs 4.27 ± 2.39, compared with scalp sham electrical stimulation; *P* = .0023), but there was no difference between the 2 groups on the other 2 functional scales, Rankin and Basel. Therefore, the authors conclude that the study supports further testing of scalp electroacupuncture for stroke, as well as further mechanistic studies to understand the mechanisms associated with the observed improvements.^[55]^

A RCTs in 2023 observed the effect of acupuncture on neurological function and serum inflammatory factor levels in patients with AIS thrombolysis. The authors reported that compared with the non-acupuncture group, the mean values of NIHSS UNK10 in the observation group were  ± SD: (7.51 ± 5.13 vs 9.59 ± 5.41; *P* < .05), mRS score (2.02 ± 0.90 vs 2.39 ± 0.85; *P* < .05), and serum Hcy (8.39 ± 4.06 vs 11.45 ± 4.71; *P* < .05), hs-CRP (6.50 ± 3.94 vs 12.04 ± 4.93; *P* < .053), MBI score (56.27 + 9.35 vs 50.78 + 8.90; *P* < .05), the improvement of the above results was better than that of the control group. The total effective rate of the observation group was 88.2% (45/51), which was higher than that of the control group 70.6% (36/51, *P* < .05). Therefore, the authors conclude that acupuncture can promote the recovery of neurological function in patients with AIS thrombolysis, improve the ability of daily living, and may reduce the level of inflammatory factors to inhibit inflammatory response and improve cerebral ischemia–reperfusion injury.^[56]^

A 2016 double-blind RCT investigated the additional effect of acupuncture on the early comprehensive rehabilitation of functional impairment secondary to acupuncture AIS and stroke by recording patients with IS after 3 weeks of acupuncture treatment compared with no intervention treatment. The authors report that, with neurological deficits assessed by changes in NIHSS as the primary outcome, a significant interaction between time and group was observed in the repeated measures ANOVA (interaction F F = 9.33, *P* < .001), and *t* test results also showed a greater change in NIHSS scores in acupuncture-treated patients with IS UNK6T (UNK6T = −4.13, *P* < .001); swallowing function was another secondary outcome we examined, repeated measures ANOVA showed a significant interaction between time and group (interaction F F = 23.62, *P* < .001), *t* test showed a significantly higher VFSS score change in the acupuncture group than in the no-acupuncture group (T T = 3.17, *P* = .002), the recovery rate was also significantly higher in patients who received acupuncture treatment UNK3 UNK4 UNK5 (^2^ UNK6 = 4.35, p *P* = .037); The acupuncture group had a significant advantage (*P* < .001) in the improvement of scores on the Mini-Mental State Examination (MMSE), the Montreal Province’s Cognitive Assessment (MoCA) scale, and based on these findings, the authors concluded that acupuncture has an additional multiple efficacy in improving neurologic deficits, swallowing disorders, cognitive impairment, and lower extremity function in patients with IS.^[57]^

Chronic pain syndrome occurs in about half of the patients with stroke, and headache is one of the common types of chronic pain syndrome after stroke.^[58]^ In 2019, 97 patients with convalescent headache of IS were randomly divided into acupuncture group (57 cases) and western medicine group (40 cases) for treatment, and the mechanism of action of acupuncture in treating convalescent headache of IS was analyzed. The authors of this study reported that compared with the western medicine group, the VAS score and headache score of the acupuncture group were significantly improved (*P* < .05). In the acupuncture group, the contents of SP, DA and 5-HT in plasma were significantly decreased (*P* < .05). Plasma endogenous opioid peptides increased significantly in the acupuncture group (*P* < .05). The total effective rate in the acupuncture group was higher than that in the western medicine group (*P* < .01). One month after treatment, the recurrence rate in the acupuncture group was lower than that in the western medicine group (*P* < .01). The authors concluded that acupuncture could decrease plasma pain-related factors and increase endogenous opioid peptides in patients with convalescent headache of IS, which provided evidence for acupuncture treatment of convalescent headache of IS.^[59]^

A RCT in 2022 compared the clinical efficacy of electroacupuncture combined with conventional western medicine with that of conventional western medicine alone in the treatment of AIS. The authors found that the NIHSS score of patients in the electroacupuncture plus conventional western medicine group was lower than that in the western medicine alone group (mean ± SD: 3.04 ± 1.26 vs 3.73 ± 1.15; *P* < .05), and the MBI score was higher than that in the western medicine alone group (80.71 ± 1.26 vs 75.77 ± 6.43; *P* < .05). The level of plasma IL-17 in the electroacupuncture plus conventional western medicine group was lower than that in the western medicine alone group (24.86 ± 4.70 vs 28.78 ± 5.48; *P* < .05), and the level of IL-10 was higher than that in the western medicine alone group (4.90 ± 0.61 vs 4.54 ± 0.62; *P* < .05). In peripheral blood of AIS patients, the level of IL-17 is increased, but the level of IL-10 is decreased. IL-17 promotes immune inflammatory reaction, further aggravates cerebral ischemic injury, including the destruction of tight junctions and the decomposition of BBB, mediates the recruitment and infiltration of neutrophils and macrophages into ischemic brain tissue, and further damages neurons. IL-10 is the main effective factor, which can inhibit the secretion of IL-1β and TNF-α. IL-10 can inhibit the expression of matrix metalloproteinase-9, protect the integrity of BBB and alleviate inflammatory injury.^[60–62]^ Therefore, the authors concluded that electroacupuncture combined with conventional western medicine could improve the neurological function and activities of daily living in patients with AIS, and its mechanism may be related to the regulation of IL-17/IL-10 imbalance, thereby reducing the neuroinflammatory response.^[63]^

YU Nan-nan et al designed a multicenter, RCT, outcome assessor blinded trial to explore the efficacy and safety of bloodletting at hand 12 well points in the treatment of acute stroke with disturbance of consciousness. In this study, participants were divided into 2 groups: bloodletting at the 12 well points of the hand plus standard medical treatment and standard medical treatment. The results showed that at 80 minutes after bleeding, the proportion of patients with improved consciousness in the blood-letting group was greater than that in the standard drug group (*P* < .05). Global Glasgow Coma Scale (GCS) scores were not significantly different between groups at the 5 time points (*P* > .05); at the same time, there was a highly significant main effect on GCS score in the blood-letting group, with a significant group × time interaction (*P* < .05). The GCS score in the blood-letting group was significantly higher than that in the standard drug treatment group at 15, 30, 50 and 80 minutes after bloodletting (*P* < .01). The scores of Glasgow Eye Score and Glasgow Verbal Score were significantly improved at 15, 30, 50 and 80 minutes after bloodletting at hand 12 well points (*P* < .05). In the moderate subgroup, the GCS scores were significantly improved within 6 hours after the onset of the disease or more than 6 hours after bloodletting at the 12 Jing-well points (*P* < .05). Further analysis of patients with IS in the moderate subgroup showed that GCS scores were significantly higher (*P* < .05) in the hand 12 well point blood-letting group at 15, 30, 50, and 80 minutes after bloodletting. Therefore, the authors concluded that blood-letting therapy at the 12 well points of the hand is safe and can improve the level of consciousness of patients with IS. It is a safe method for emergency treatment of acute stroke.^[64]^

Twenty-five RCTs, including a total of 2224 participants, were reviewed in a meta-analysis to investigate the efficacy and safety of using acupuncture for the treatment of IS compared with medication or sham acupuncture. From the data obtained, there was a significant difference between the acupuncture treatment groups in clinical efficacy (OR = 4.04, 95% CI [2.93, 5.57], *P* < .001), Fugl-Meyer assessment (MD = 11.22, 95% CI [7.62, 14.82], *P* < .001), Barthel index score (MD = 12.84, 95% CI [9.85, 15.82], *P* < .001) and neurological deficit score (MD = −2.71, 95% CI [−3.84, −1.94], *P* < .001) were better than those in the control group. While most of the results included in this analysis support acupuncture, the authors conclude that further studies are necessary to determine the results due to the low to very low quality of evidence for the RCT results included in the meta-analysis; The authors do, however, conclude that acupuncture is superior to non-acupuncture or drug therapy in the treatment of IS.^[65]^

Several meta-analyses have also attempted to better characterize the efficacy and safety of the use of electroacupuncture for the treatment of AIS. A 2015 meta-analysis studied 18 RCTs with 1411 participants to investigate the effectiveness of electroacupuncture compared to conventional treatment and sham acupuncture. The results of meta-analysis showed that the electroacupuncture treatment group was superior to the conventional western medicine treatment group in improving Barthel index (*P* < .01), Fugl-Meyer score (*P* < .01), NIHSS score (*P* < 0. 01), and revised Scandinavian Stroke Scale (*P* < 0. 01). In the analysis of total clinical effective rate, there was a significant difference between electroacupuncture and conventional western medicine treatment (*P* < .01). The authors conclude that while these findings support the evidence for electroacupuncture in AIS, further large-scale RCTs are needed to validate these findings.^[66]^

While most of the papers reviewed here demonstrate the efficacy of acupuncture in the treatment of IS, it is acknowledged that the heterogeneity of the statistical analysis, the study population (e.g., sample size), and the treatment method (e.g., length of acupuncture treatment, acupoint changes) make it difficult to draw definitive conclusions. More high-quality evidence is still needed to compare acupuncture with other treatment modalities: control, sham acupuncture, acupoint catgut embedding, drug therapy, etc.^[65,66]^

Finally, a 2021 study investigated the effect of acupuncture on functional connectivity between the hemispheres and regional brain regions in patients with dominant MCA AIS, and showed that the functional connectivity between bilateral BA6 and BA8 dominated brain regions was enhanced after combined scalp acupuncture and western medicine treatment compared with western medicine treatment alone. BA6 and BA8 form the premotor cortex. The values of ALFF and ReHo in the left angular gyrus (BA39) and middle temporal gyrus were positively correlated with AIS. The values of ALFF in the superior temporal gyrus of the dominant hemisphere were increased. The superior temporal gyrus, angular gyrus, and middle temporal gyri were jointly responsible for auditory and motor integration. ReHo values were increased in the fusiform gyrus (BA37), superior limbic gyrus (BA40), and BA7. BA37 is an important part of object category recognition, and BA40 is an important center responsible for fine motor coordination, complex movement and labor skills. BA7 combines visual and motor information to perform visual–motor coordination functions. These data suggest that scalp acupuncture treatment can enhance regional brain functional activity and functional connectivity between hemispheres related to sensory integration, language processing, and motor coordination in patients with MCA AIS.^[67]^

## 6. Conclusion

Acupuncture is one of the most commonly used treatment techniques in TCM, which is a process of treating diseases by stimulating the skin or deep tissues at specific locations (acupoints) of the body. Acupuncture and moxibustion choose different stimulation methods, or choose different acupoints for stimulation, which will affect nerve regeneration in different areas of the brain.^[68]^ While modern researchers are recognizing the fact that IS is one of the most disabling cerebrovascular diseases in the world, clinicians and researchers are gaining a clearer understanding of the pathophysiology of IS and are looking for clinically effective treatments with minimal side effects. Therefore, although the main treatment of IS is thrombolysis and endovascular therapy, acupuncture has a positive impact on analgesia, limb rehabilitation, improvement of peri-infarct blood flow and stimulation of cerebral lobes with low blood flow perfusion, and neuronal reorganization in patients with IS. Therefore, acupuncture has attracted more and more attention as a feasible supplement to the later treatment management of patients with IS. Many studies have shown that acupuncture is a safe, useful and available alternative therapy that may be beneficial for some patients with IS. However, further large-scale RCTs are necessary to further consolidate these findings and provide further support for the clinical value of acupuncture.

Although previous studies have analyzed the effects of acupuncture on IS, further investigation is still needed to ensure that the inclusion of acupuncture in the therapeutic management of IS results in positive outcomes for patients. At the same time, there is no uniform quantitative standard for the treatment method of acupuncture and moxibustion, and the interval and duration of treatment are different. There are also many disputes among experts on acupoint selection, acupuncture manipulation, stimulation intensity, and needle retention time, which need to be further quantified and standardized. Future studies could also benefit from regular follow-up treatment and interviews.

## Author contributions

**Conceptualization:** Haishen Zhao.

**Data curation:** Manya Wang.

**Formal analysis:** Zuoshan Wang.

**Funding acquisition:** Zuoshan Wang, Manya Wang.

**Investigation:** Zuoshan Wang.

**Methodology:** Zuoshan Wang.

**Project administration:** Zuoshan Wang, Manya Wang.

**Supervision:** Manya Wang.

**Validation:** Manya Wang.

**Visualization:** Manya Wang.

**Writing – original draft:** Zuoshan Wang.

**Writing – review & editing:** Zuoshan Wang.
